# Impacts of Flow Rate and Pulsed Electric Field Current Mode on Protein Fouling Formation during Bipolar Membrane Electroacidification of Skim Milk

**DOI:** 10.3390/membranes10090200

**Published:** 2020-08-26

**Authors:** Vladlen S. Nichka, Thibaud R. Geoffroy, Victor Nikonenko, Laurent Bazinet

**Affiliations:** 1 Department of Food Sciences, Laboratoire de Transformation Alimentaire et Procédés ÉlectroMembranaires (LTAPEM, Laboratory of Food Processing and Electromembrane Processes), Institute of Nutrition and Functional Foods (INAF), Dairy Research Center (STELA), Université Laval, Québec, QC G1V 0A6, Canada; vladlen.nichka.1@ulaval.ca (V.S.N.); thibaud.geoffroy.1@ulaval.ca (T.R.G.); 2 Physical Chemistry Department, Kuban State University, 149 Stavropolskaya str., 350040 Krasnodar, Russia; v_nikonenko@mail.ru

**Keywords:** electrochemical acidification, electrodialysis, casein, concentration polarization, ion-exchange membrane, fouling, Reynolds number, mode of current, flow flush

## Abstract

Fouling is one of the major problems in electrodialysis. The aim of the present work was to investigate the effect of five different solution flow rates (corresponding to Reynolds numbers of 162, 242, 323, 404 and 485) combined with the use of pulsed electric field (PEF) current mode on protein fouling of bipolar membrane (BPM) during electrodialysis with bipolar membranes (EDBM) of skim milk. The application of PEF prevented the fouling formation by proteins on the cationic interface of the BPM almost completely, regardless of the flow rate or Reynolds number. Indeed, under PEF mode of current the weight of protein fouling was negligible in comparison with CC current mode (0.07 ± 0.08 mg/cm^2^ versus 5.56 ± 2.40 mg/cm^2^). When a continuous current (CC) mode was applied, Reynolds number equals or higher than 323 corresponded to a minimal value of protein fouling of BPM. This positive effect of both increasing the flow rate and using PEF is due to the facts that during pauses, the solution flow flushes the accumulated protein from the membrane while in the same time there is a decrease in concentration polarization (CP) and consequently decrease in H^+^ generation at the cationic interface of the BPM, minimizing fouling formation and accumulation.

## 1. Introduction

Bovine milk is one of the most important raw materials in the food industry, which is composed of water, lactose, lipids, proteins, and minerals [1]. Caseins are the main proteins of milk; their contents are around 80% of total proteins [2]. Caseins are of great interest due to their nutritional value and functional properties. These proteins are used in processed cheese, coffee whiteners, infant formulas, and in pharmaceutical products [3]. They are also used in the manufacture of paper coatings, textile fabrics, adhesives, concrete, paints, and cosmetics [4]. One of the ways of casein production from milk is using chemical acidification. However, this method has drawbacks such as producing large amounts of salts, which have to be separated from the acid whey resulting in undesired waste streams [5,6].

Bazinet et al. [7] developed a different method of acid casein production using electrodialysis with bipolar membranes (EDBM) of milk. EDBM combines conventional electrodialysis with the special properties of bipolar membranes (BPM) to split water with the protons leading to protein precipitation while the selectivity of the monopolar membranes allows demineralization of the final acid whey. The major problems during EDBM are protein fouling and scaling of membranes. Scaling and colloidal fouling lead to an increase in electrical resistance, a decrease in permselectivity and membrane alteration [8,9]. The presence of fouling significantly increases the cost of electrodialysis in the food industry. According to Mikhaylin and Bazinet [10] the costs of membrane regeneration and replacement amount from 20–30% (for pressure driven processes) to 40–50% (for electrically driven processes) of the total costs for the membrane processes in food industry.

There are different ways to prevent or minimize fouling formation during ED treatment. It could be membrane modification, cleaning procedures, pretreatment (for example with pressure-driven processes), mechanical actions, ED with reverse polarity, overlimiting current regimes and others [10]. All of these methods have different disadvantages and operations limitations. Using these methods can lead to additional expenses, membrane deterioration, generation of additional effluents, or they are not suitable with ED systems where BPMs are stacked. A recent effective way to prevent or minimize fouling formation is the use of pulsed electric fields (PEF) [11,12,13]. In this non-stationary current mode, continuous current (CC) pulses alternate with pauses during which the current is equal to zero. The positive effect of PEF mode of current is associated with a decrease in CP phenomenon and hence a decrease in water splitting and an increase in ED power efficiency [14]. Indeed, during the pause lapse, when there is no water splitting, the ion concentration at the membrane interfaces can be partially restored, reducing the CP phenomena and membrane fouling during the subsequent pulse lapse [15].

It was also demonstrated that an optimization of hydrodynamic conditions during electrodialysis can also help to minimize the membrane fouling and scaling formation. The influence of flow rate on the electroacidification parameters (duration of the process, anode/cathode voltage difference and conductivity of milk) was previously investigated by Bazinet et al. [7] during EDBM of skim milk for bovine casein production, but the authors did not study the effect of current mode on the process. In the paper of Mikhaylin et al. [16] authors observed that the use of higher flow rates during EDBM of skim milk coupled with an ultrafiltration module leads to more than 38% decrease in CEM scaling formation in comparison to the conventional treatment due to the creation of unfavorable hydrodynamic conditions for the scaling attachment and growth, but the authors did not study protein fouling due to the use of UF module prior EDBM. Increasing of flow rate has similar effect in the ED cell as the use of PEF. It has two main advantages: decrease of water splitting rate and prevention of fouling formation due to the higher mixing of solution during the process. The decrease in the rate of water splitting is due to the decrease in the diffusion layer thickness (decrease of CP phenomenon) caused by the increase of the flow rate [17]. However, the coupled effect of mode of current supply and hydrodynamic conditions on the protein fouling formation has never been studied before.

In this context, the aim of the present study was to evaluate the influence of solution flow rate coupled with the mode of current (CC and PEF) on the protein fouling formation on BPM during EDBM of skim milk. The specific objectives of the work were: (1) to study the impact of different flow rates on protein fouling at the interface of the BPM; (2) to test the effect of PEF on protein fouling formation and to compare the results with those in CC mode; (3) to characterize the membrane properties before and after EDBM; (4) to link the protein fouling formation with the hydrodynamic condition in the milk channel; and (5) to propose mechanisms for protein fouling mitigation in the different conditions tested.

## 2. Materials and Methods 

### 2.1. Materials

The milk used in this study was a commercial homogenized and pasteurized skim milk Beatrice (Parmalat, Victoriaville, QC, Canada). Sodium sulphate (Na_2_SO_4_, ACS grade) and potassium chloride (KCl, ACS grade) were obtained from BDH (VWR International, Mississauga, ON, Canada). Chemicals for cleaning of the electrodialysis (ED) system, hydrochloric acid (HCl) and sodium hydroxide (NaOH), were purchased from Fisher Scientific (Thermo Fisher Scientific, Montreal, QC, Canada). The average concentrations of the milk were determined using a Delta Lactoscope FTIR dairy analyzer (Delta Instruments, Drachten, Netherlands). The milk composition is presented in Table 1. This composition is consistent with data in the literature [18].

### 2.2. Methods

#### 2.2.1. Electrodialysis Cell

**Figure 1:**
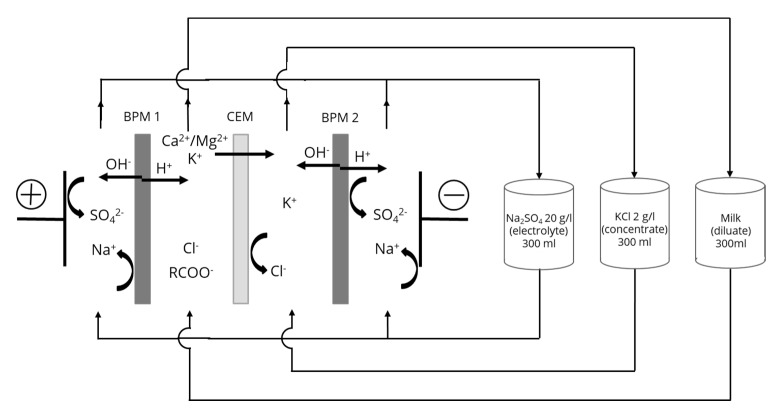
Electrodialysis cell configuration of electrodialysis with bipolar membranes (EDBM) process.

The electroacidification cell was a Microflow-type cell (Electro-Cell AB, Täby, Sweden) consisting of four compartments separated by one Neosepta cationic membrane (CMX-fg) and two Neosepta bipolar membranes (BP-1E) (Astom, Tokyo, Japan) (Figure 1). The membranes tested had an effective surface area of 16 cm^2^. The anode was a plate dimensionally stable electrode (DSA-O_2_, Ti/IrO_2_ coating) and the cathode a 316-stainless-steel electrode. This arrangement defines three closed loops containing equal volumes (300 mL) of the 20 g/L Na_2_SO_4_ electrolyte solution, 2 g/L KCl aqueous solution and the milk solution. The flow rates were equal to the flow rate tested for each solution used in the experiment. Each closed loop was connected to a separate external plastic tank, allowing a continuous recirculation. The ED system was not equipped to maintain a constant temperature, but since this parameter underwent low variations (between 25 and 37 °C) similar for each mode of current, the temperature was not further discussed.

#### 2.2.2. Protocol

EDBM was carried out using a constant current intensity of 50 mA (corresponding to a current density of 3.13 mA/cm^2^) generated by using a Xantrex power supply, model HPD 60-5SX (Xantrex Technology Inc., Burnaby, BC, Canada). ED experiments were performed using CC or PEF with 10 s pulse and 50 s pause durations. In addition, for each mode of current, five different flow rates were investigated (400, 600, 800, 1000, and 1200 mL/min) corresponding to Reynolds numbers of 162, 242, 323, 404, and 485 to test the combined effect of current mode and flow rate on protein fouling during EDBM of skim milk. Three replicates of each condition were performed in this experiment. PEF was generated by a modified Bio-Rad Pulsewave 760 generator (Bio-Rad laboratories, Richmond, BC, Canada). In this study, the conventional ED experiment under CC regime was considered as the control. Conventional ED experiment was stopped after 20 min whereas for PEF the duration of experiment was 120 min in order to compare the different current mode with respect to the same amount of charges transported.

#### 2.2.3. Solution Conductivity

An YSI conductivity meter, model 3100 was used with an YSI immersion probe, model 3252, cell constant K = 0.1 cm^−1^ (Yellow Springs Instrument Co., Yellow Springs, OH, USA) to measure the conductivity of the milk and KCl solutions.

#### 2.2.4. Membrane Thickness and Electrical Conductivity

The membranes were soaked in a 0.5 M NaCl solution for 30 min before and after each ED experiment for their characterization. The membrane thickness was measured using Marathon electronic digital micrometer, (Marathon watch company LTD, Richmond Hill, ON, Canada). The micrometer was equipped with a 10-mm-diameter flat contact point. The membrane thickness value was measured at six different locations on the effective membrane surface and then averaged [19]. The electrical conductivity of the membrane was calculated from the measured thickness and its electrical resistance, obtained from the membrane conductance. The conductance was measured using an YSI conductivity meter, model 3100 (Yellow Springs Instrument Co., Yellow Springs, OH, USA) equipped with a specially designed clip as described by Cifuentes-Araya [19]. A 0.5 M NaCl solution was used as a reference solution. Six conductance measurements of the reference solution and of the membrane in the reference solution were taken. The membrane conductance in the reference solution was taken on the effective membrane surface. Membrane conductivity (*k*) was calculated according to Equation (1) [20]. In Equation (1) *k* is the membrane electrical conductivity (in S/cm), *L* the thickness of the membrane (in cm) and *A* the electrode area (1 cm^2^):

(1) $$k=\frac{L}{R_{m}A}$$

The membrane electrical resistance (*R_m_*) was calculated according to Equation (2). In Equation (2) *G_m_* is a membrane conductance:

(2) $$R_{m}=\frac{1}{G_{m}}$$

Membrane resistance (*R_m_*) was obtained by subtracting the solution resistance (*R_s_*) to the membrane resistance soaking in the solutions (*R_s+m_*).

#### 2.2.5. pH of the Diluate and Concentrate

The pH of milk was measured with a VWR Symphony SP70P pH-meter (VWR International, Montreal, QC, Canada). The pH of KCl solution was measured with an Thermo Scientific Orion Star A221 pH-meter (Thermo Fisher Scientific, Montreal, QC, Canada).

#### 2.2.6. Fouling Weight

Fouling was collected from the BPM surface, and then freeze-dried in a Labconco Lyophyliser, model Freezone 4.5 (Labconco Corporation, Kansas City, MO, USA). After weighing the dried powders, they were kept at −20 °C until further analyses.

#### 2.2.7. Membrane Surface Photographs

Digital photographs of the BPM cationic interface in contact with milk were taken after each EDBM experiment.

#### 2.2.8. Number of Charges Transported

The number of charges transported was calculated according to Equation (3). In Equation (3) *Q* is the number of charges transported (in C), *I* is the current intensity (in A), *t* is the duration of the experiment (in s).

(3) Q=It

When using PEF, *I* was constant during the pulse period (the same as in the CC mode), and zero during the pauses.

#### 2.2.9. Energy Consumptions

The energy consumptions (in Wh) was calculated according to Equation (4). In equation (4), *I* is the current intensity (in A), *U*(*t*) the voltage (in V) as a known from experiment function of time [19]. The time taken into account for the PEF mode of current was the effective time during the pulse periods.

(4) $$EC=I\int^{\;}U\left(t\right)dt$$

#### 2.2.10. Reynolds Numbers

Reynolds number was calculated according to Equation (5) in order to evaluate the flow motion in a system. In Equation (5), ρ is the density of the fluid (in kg/m^3^), ν is the average flow velocity (in m/s), *h* is the intermembrane distance (in m), *µ* is the dynamic viscosity of the fluid (in Pa s) [21].

(5) Re=ρνhμ

Density ρ as well as dynamic viscosity *µ* of skim milk are known parameters, which were taken equal to 1035 kg/m^3^ and 0.0015 Pa s respectively.

#### 2.2.11. Statistical Analyses

All data were subjected to a two-way or three-way analysis of variance (ANOVA) using SigmaPlot software (SigmaPlot version 12.5, Chicago, IL, USA). Fisher’s Least Significant Difference (LSD) test was used to determine the effect of each factor under study on fouling kinetics.

## 3. Results and Discussion

### 3.1. Evolution of PH

#### 3.1.1. In Skim Milk

**Figure 2:**
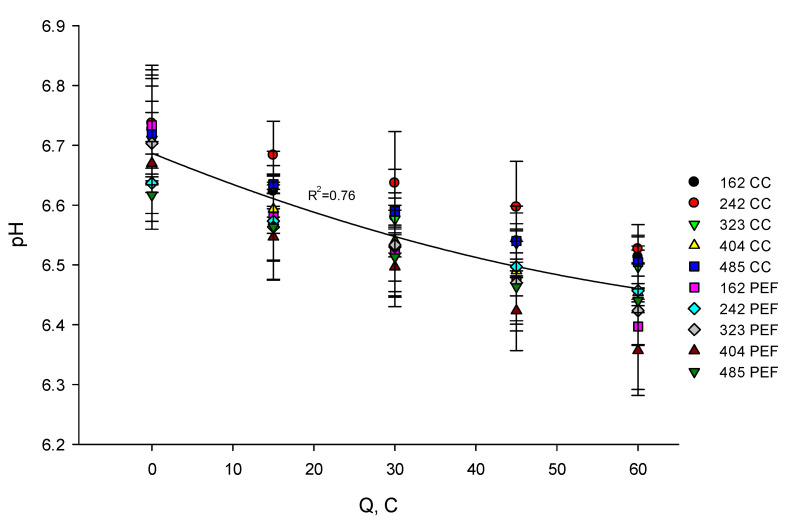
Evolution of milk pH as a function of the number of charges transported (in Coulomb) during EDBM carried out in different conditions of flow rate (corresponding to indicated Reynolds numbers) and current mode. Reynolds numbers of 162, 242, 323, 404 and 485 correspond to flow rates of 400, 600, 800, 1000 and 1200 mL/min respectively. CC—constant current and PEF—pulsed electric field.

It appeared from the statistical analysis, that the mode of current supply and flow rate had no significant effect (*p* > 0.05) on the variation of pH of the skim milk during EDBM. The regression curve calculated for the pH of milk as a function of number of charges transported (Figure 2) showed a decrease in pH of 0.23 ± 0.06 per C for all cases considered (3.4% decrease). The decrease in pH of milk can be explained by addition of H^+^ which occurs by splitting of water molecules at the BPM cationic interface during electrochemical acidification of milk. The slow acidification of milk can be explained by its buffer capacity (i.e., proteins, weak acids) and the release of phosphate anions from the casein micelles, which neutralize the acidification effect of the H^+^ addition [22]. Indeed, with decrease of pH, protein-bounded calcium (or magnesium) phosphates (or citrates) compounds convert to the soluble ionic form and remain in the whey fraction of milk [23]. Numerous papers proved the fact that the decrease in pH of milk leads to dissolving colloidal calcium phosphate and small amounts of magnesium [24,25,26] and causes the dissociation of casein from micelles [27]. Milk pH decreased in a similar way to the one observed previously by Bazinet et al. [28] and Masson et al. [29] but with a different cell design (effective surface area of 100 cm^2^).

#### 3.1.2. In KCl Solution

**Figure 3:**
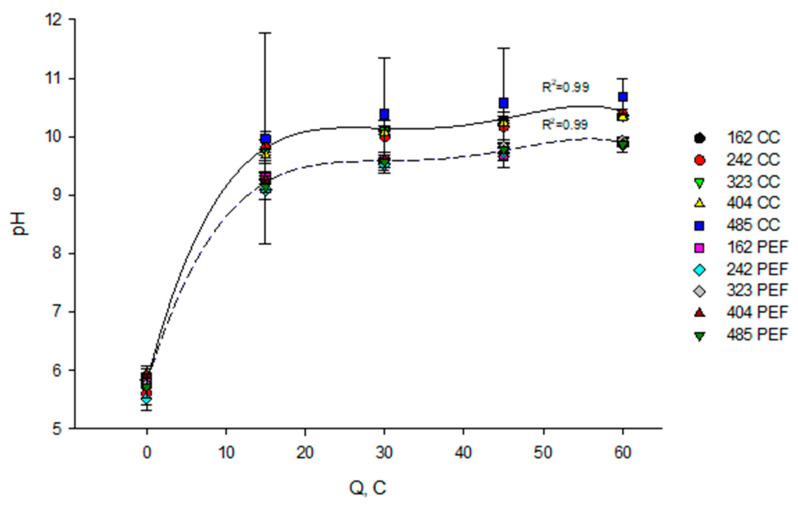
Evolution of KCl solution pH as a function of the number of charges transported (in Coulomb) during EDBM carried out in different flow rates (corresponding to indicated Reynolds numbers) and current modes (PEF in dashed line and CC in solid line). Reynolds numbers of 162, 242, 323, 404 and 485 correspond to flow rates of 400, 600, 800, 1000 and 1200 mL/min respectively. CC—constant current and PEF—pulsed electric field.

The analysis of variance showed that the mode of current (*p* < 0.001) and flow rate (*p* = 0.02) have a significant effect on the variation of pH of the KCl solution during EDBM while the coupled effect of flow rate/mode of current has no effect (*p* = 0.92). Coupled effect represents the combined effects of both factors (flow rate and current mode) on the dependent measure (pH of KCl). Based on the *p* value it can be concluded that the current mode has the main effect on the pH of KCl. The conclusion that flow rate is a significant factor and coupled effect of flow rate/mode of current is not significant was mainly due to the high standard deviation of experimental data under CC mode and highest flow rate (which corresponds to a Reynolds number of 485), for other conditions the standard deviations were significantly small. Considering that, there was a major effect of current mode but the flow rate applied, and the resulting Reynolds number, for a mode of current did not influence on the variation of pH of the KCl solution. The curve of pH evolution calculated for the KCl solution as a function of number of charge transported (Figure 3) showed an increase in pH, which is different depending of the mode of current (CC vs PEF). Hence, whatever the flow rate of KCl solution, in the case of CC, the pH increased from 5.8 to 10.4 (79.3% increase), while it increased to 9.9 (70.7% increase) in the case of PEF. It is probably connected with the fact that during the pause lapse under PEF occurs a better mixing of the solution and equilibration allowing phosphate from micelle to better dissolve. The lower final pH of KCl solution under the PEF can also be connected with a leakage of OH^−^ through CMX membrane during a pause lapse [30] and decreasing of CP phenomenon and consequently with a decrease in water splitting [14]. Indeed, during the pause lapse of PEF, the ion concentration at the membrane interface can be partially restored, reducing the CP phenomena and membrane fouling during the subsequent pulse [31]. The difference in pH between CC and PEF appeared at the beginning of the process (up to 15 C) and then this difference remained constant until the end of experiment. Rapid increase of pH of the KCl solution at the beginning of EDBM occurred as the hydroxide electrogeneration progresses due to the water splitting at the BPM. Furthermore, the variation of pH is more important in the KCl solution that in the milk due to the different buffer capacities of the two solutions. The KCl has no buffer capacity while milk according to its composition has a high buffer capacity; phosphate, citrate, lactate, carbonate, acetate, and propionate ions are mainly responsible for the buffer capacity of milk [32].

### 3.2. Evolution of Conductivity

#### 3.2.1. In Skim Milk

**Figure 4:**
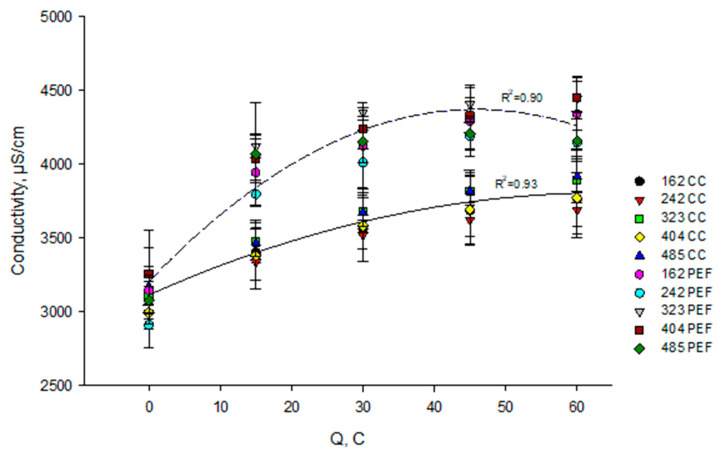
Evolution of milk conductivity as a function of a number of charges transported (in Coulomb) during EDBM carried out in different conditions of flow rate (corresponding to indicated Reynolds numbers) and current mode (PEF in dashed line and CC in solid line). Reynolds numbers of 162, 242, 323, 404 and 485 correspond to flow rates of 400, 600, 800, 1000 and 1200 mL/min respectively. CC—constant current and PEF—pulsed electric field.

It appeared from the statistical analysis, that the mode of current has a significant effect (*p* < 0.001) on the variation of skim milk conductivity during EDBM while the flow rate and the coupled effect of flow rate/mode of current have no effect (*p* > 0.05). The regression curves calculated for the milk conductivity as a function of number of charge transported (Figure 4) showed an increase in conductivity, different according to the mode of current (CC vs PEF). Hence, whatever the flow rate of the milk solution, in the case of CC, the electrical conductivity increased from 3100 to 3800 μS/cm (22.5% increase), while it increased to 4300 μS/cm (38.7% increase) in the case of PEF. The difference in electrical conductivity between both modes of current appeared at the beginning of the process (up to 15 C) and then it was quite constant until the end of the experiments. The conductivity changes of skim milk at a given pH is the result of H^+^ generation, change in global soluble protein charge, dissolving of calcium, phosphate and magnesium from casein micelles, as well as potassium, sodium, chloride, hydrogen ions, and citrate already present in the soluble phase [33]. Indeed, according to [25,31] Ca^2+^ and Mg^2+^ are bound to the phosphoserine groups of different individual caseins of milk and remain as colloidal phosphocalcic (phosphomagnesic) bridges inside the casein micelles and are released as pH decreases which influences the resulting milk conductivity. Generally, electrical conductivity of milk was influenced by two competing phenomena: desalination and acidification. Cations migrate from the desalination channel by the action of CEM, which diminished the conductivity of solution. On the other hand, the hydrogen ions generated on the cationic interface of BPM and breakage of casein micelles contribute to the conductivity of milk. The increase of milk conductivity was probably connected to the release of calcium ions from casein micelle, which acts opposite to the demineralization effect and the rate of calcium release is higher than its migration through the CEM since many potassium ions are present in the solution. Indeed, it was reported by Bazinet et al. [34] that potassium is the first ion to migrate during EDBM, due to its higher electrical mobility, concentration and also the predominant one to leave the skim milk solution until a critical concentration of about 20% of its initial concentration was reached. A faster increase in electrical conductivity is observed at the beginning of EDBM under the PEF current mode. This would be linked to the fact that, under CC, electrogenerated H^+^ would interact preferentially with phosphate ions released from casein micelle and consequently, these protons, would not contribute to the increase in milk conductivity. Indeed, previous works have already showed a decrease in milk conductivity during EDBM due to the demineralization effect of CEM [25,30,31].

#### 3.2.2. In KCl Solution

**Figure 5:**
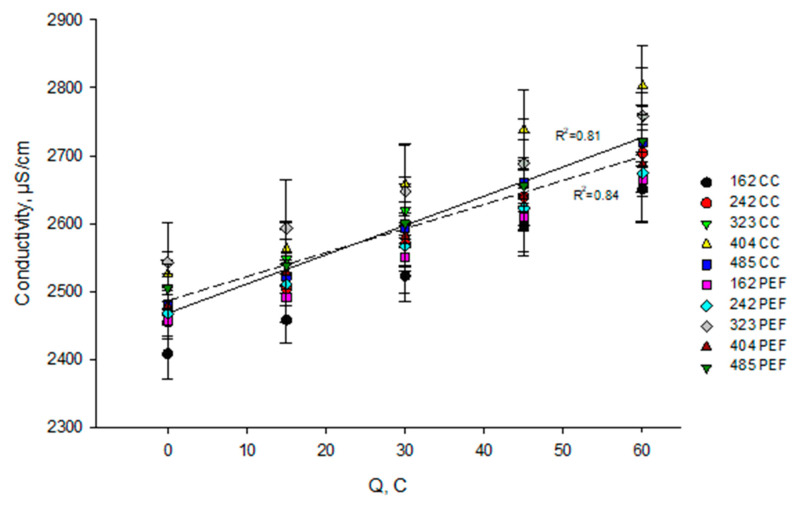
Evolution of KCl compartment conductivity as a function of the number of charges transported (in Coulomb) during EDBM carried out in different conditions of flow rate (corresponding to indicated Reynolds numbers) and current mode (pulsed electric field (PEF) in dashed line and continuous current (CC) in solid line). Reynolds numbers of 162, 242, 323, 404, and 485 correspond to flow rates of 400, 600, 800, 1000, and 1200 mL/min respectively. CC—constant current and PEF—pulsed electric field.

The analysis of variance showed that the mode of current has a significant effect (*p* < 0.001) on the variation of KCl solution conductivity during EDBM while the flow rate and the coupled effect of flow rate/mode of current has no effect (*p* > 0.05). Hence, the electrical conductivity of the KCl solution increased for both current modes but differently and can be approximated linearly (Figure 5). Indeed, the variations of conductivity between the beginning and the end of EDBM, whatever the flow rate of solution, were of 251.5 mS/cm (corresponding to a 10.2% increase) and 211.0 mS/cm (8.4% increase) for CC and PEF respectively. Although there was a statistical difference, which was relatively small, it corresponds to less than 1.7% of the initial conductivity. Consequently, the evolution of KCl conductivity can be considered as the same and that whatever the flow rate and the current mode. Unlike the electrical conductivity of milk, for the KCl solution both process electrically-driven calcium, magnesium, and potassium ion migration and electrogeneration of hydroxide ions on the BPM surface acted together and lead to such an increase in conductivity during EDBM. Indeed, Lin Teng Shee et al. [35] have reported the fact that the H^+^ and OH^−^ produced during the process of solution demineralization using BPMs contribute more to the conductivity than other ions. The same effect of increase of KCl conductivity was observed by Kravtsov et al. [36] for acid whey demineralization using EDBM. In the case of CC mode, a faster increase of the KCl solution conductivity was observed compared to a PEF current mode. It was connected with a more intensive water splitting phenomenon under CC current mode as we mentioned previously for pH evolution of the KCl solution.

### 3.3. Membrane Parameters

According to the statistical analysis of the difference between membrane conductivity before and after experiments (*p* < 0.001, *p* < 0.001 and *p* = 0.003 for CEM, BPM1, and BPM2 respectively), all the membranes showed a decrease in conductivity after treatment (Table 2). It also appeared from the analysis of variance that flow rate has no significant effect (*p* > 0.05) on the conductivity variation of membranes after EDBM. For the CEM and BPM1 the mode of current has no significant effect on the conductivity (*p* > 0.05) while in the case of BPM2 this factor has a significant effect (*p* = 0.04) on the conductivity variations. The conductivity of CEM decreased probably due to the substitution of relatively mobile Na^+^ by Ca^2+^ from milk [37]. Electrical conductivity of the first BPM decreased due to the presence of protein fouling on the cationic interface. This is known fact that the fouling and scaling formation decrease the conductivity and permselectivity of membranes [8,9]. Despite the fact that ANOVA showed that there was a significant difference of BPM2 conductivity after treatment in general and for different modes of current in particular we can observe that all the values are in the range of standard deviation. Indeed, an average conductivity of BPM2 before treatment is 7.45 ± 0.17 mS/cm, after is 7.15 ± 0.26 mS/cm for all the conditions averaged. If we compare different modes of current, we can notice that under CC the average conductivity of BPM2 is 7.30 ± 0.26 mS/cm, under PEF is 7.01 ± 0.24 mS/cm. Thus, we can conclude that the difference of conductivity of BPM is not significant before and after EDBM treatment.

Concerning the thickness of membranes, it was concluded that there was no significant difference in CMX thickness before and after experiments (*p* > 0.05) (Table 2). The averaged thickness of the CMX membranes was 0.149±0.003 mm. According to the ANOVA, the thickness of both BPMs significantly changed after all experiments (*p* < 0.001, *p* = 0.002 for BPM1 and BPM2 respectively) but the values did not depend on the flow rate (*p* > 0.05) and current mode (*p* > 0.05) during EDBM. Despite the fact that the changes of BPMs thicknesses are significant after EDBM we can notice that these values are in the range of standard deviation; BPM1 thickness before treatment is 0.246 ± 0.002 mm and 0.242 ± 0.002 mm after and for BPM2 0.245 ± 0.002 mm and 0.241 ± 0.002 mm respectively. Since that there is no real difference in thickness of the BPMs before and after EDBM treatment. The thickness of BPM in contact with milk did not change after treatment despite the presence of protein fouling for some cases due to the fact that fouling was removed from the membrane surface before thickness measurements.

### 3.4. Membrane Surface Integrity and Quantification of Protein Fouling 

**Figure 6:**
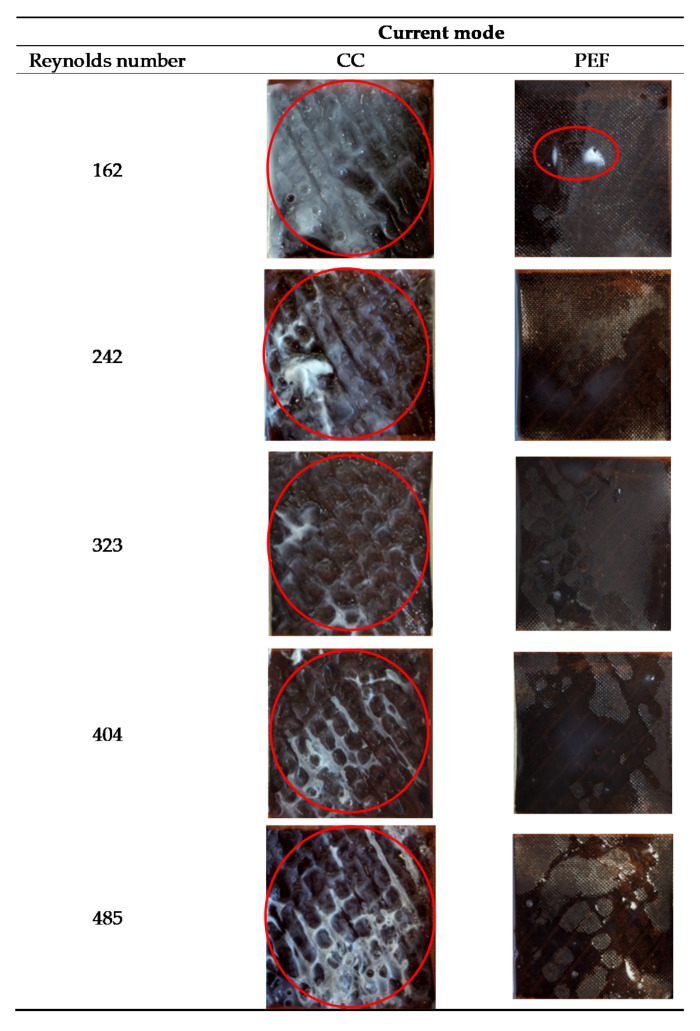
Photographs of the bipolar membrane (BPM) cationic interface in contact with milk after EDBM carried out in different flow rates (corresponding to indicated Reynolds numbers) and current modes (CC and PEF). The red circles indicate a fouling.

Concerning the CEM for both interfaces considered, as well as for the anionic side of the BPM, no fouling was observed whatever the conditions of flow rate and current mode applied. In contrast, the photographs of the cationic interface of the BPMs, in contact with the milk solution, showed that there was a major impact of current mode on the presence or not of a protein fouling at this interface while flow rate was less impacting (Figure 6).

**Figure 7:**
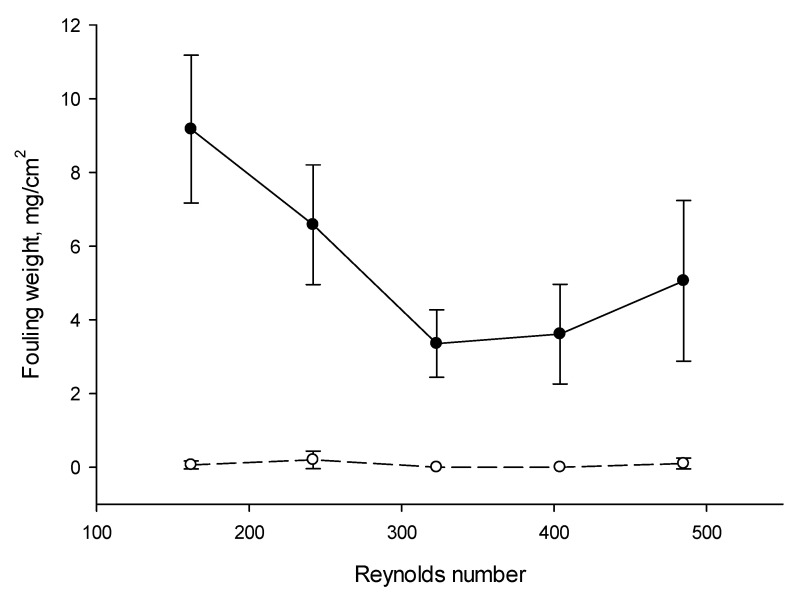
Weight of protein fouling (in mg/cm2) recovered on the BPM cationic interface in contact with milk after EDBM depending on the Reynolds numbers under CC and PEF current mode (PEF in dashed line and CC in solid line). Reynolds numbers of 162, 242, 323, 404 and 485 correspond to flow rates of 400, 600, 800, 1000 and 1200 mL/min respectively. CC—constant current and PEF—pulsed electric field.

Considering the weight of protein fouling recovered on each cationic interface after EDBM treatment, the ANOVA showed that there is an effect of current mode (*p* < 0.001), flow rate (*p* = 0.005) as well as coupled effect mode of current/flow rate (*p* = 0.006). Indeed, with PEF mode of current the weight of protein fouling was quite negligible in comparison with CC with values, all flow rate conditions averaged, of 0.07 ± 0.08 mg/cm^2^ and 5.56 ± 2.40 mg/cm^2^ respectively. Confirmation of the positive impact of using PEF current regimes on preventing of protein fouling and scaling formation can be found in many papers [11]. In the work of Ruiz et al. [11] it was also demonstrated that PEF with 10–40 s pulse/pause combination whatever the conditions allowed to completely eliminate protein fouling from the AEM during demineralization by conventional ED of a casein model solution. The protein fouling formation under CC mode was observed because of a local pH change at the BPM interface, due to overlimiting conditions, since only small changes in the pH of the skim milk were observed during the treatment; the pH of the milk solution bulk was always over the isoelectric point (pH 4.6). Positive effects of using PEF for fouling minimization were explained by the fact that the PEF produces perturbations in electrophoretic movement of the substances forming the screening film on the surface of membrane [14]. These perturbations increase the mixing of solution within the boundary layer and hinder the formation of deposit at the membrane surface. The impact of flow rate has already been demonstrated by Bazinet et al. [7] on milk acidification but at only two different flow rate conditions (757 mL/min and 4542 mL/min) and with a different cell design (effective surface area of 100 cm^2^). However, the impact of flow rate coupled with PEF has never been demonstrated before. Concerning flow rate, it has no impact during application of PEF with very low or no quantity of protein fouling while during CC, the weight of fouling was different according to the flow rate applied. Indeed, for CC, there was an almost linear decrease of the weight of protein fouling as a function of Reynolds numbers from 162 to 323 (which corresponds to a flow rate from 400 to 800 mL/min for the ED system used) since it decreased from 9.18 to 3.36 mg/cm^2^ (Figure 7). A further increase in flow rate up to the Reynolds numbers of 485 did not lead to significant changes in the amount of fouling formed on the BPM surface. 

According to literature data [38], all the Reynolds numbers reached in the present work correspond to a steady laminar fluid flow in the channel, while the turbulent fluid flow regime begins when the Reynolds numbers reach amount of 1000. Consequently, the decrease in the amount of protein fouling on the BPM surface with an increase of the Reynolds number up to value of 323 was observed due to the creation of unfavorable hydrodynamic conditions for the fouling attachment and growth. The similar effect for scaling formation was observed by Mikhaylin et al. [17] where authors showed that the use of higher flow rates during BMEA of skim milk coupled with ultrafiltration (UF) module leads to more than 38% decrease in scaling in comparison to the conventional EDBM-UF treatment. However, they did not mention the impact on protein fouling since they used a UF membrane to prevent such a fouling inside the EDBM cell. The positive coupled effect of PEF and flow rate on the fouling formation is due to the fact that during pauses the excess of H^+^ ions at the cationic interface of the BPM, initiating the surface fouling, is dissipated due to the recirculation of solution and since no more H^+^ are generated, avoiding consequently the fouling formation and accumulation while in the same time the solution flow flushes the potentially accumulated protein from the membrane surface.

### 3.5. Energy Consumptions 

**Figure 8:**
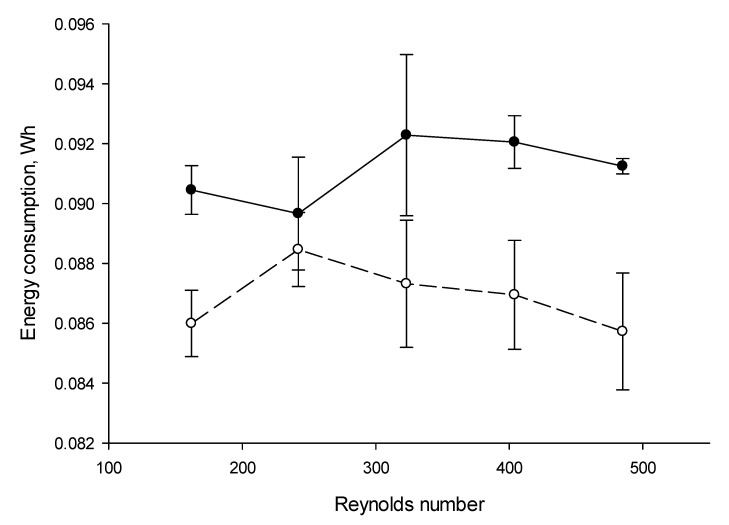
Energy consumptions (in Wh) during EDBM depending on the Reynolds numbers under CC and PEF current mode (PEF in dashed line and CC in solid line). Reynolds numbers of 162, 242, 323, 404, and 485 correspond to flow rates of 400, 600, 800, 1000 and 1200 mL/min respectively. CC—constant current and PEF—pulsed electric field.

It appeared from the statistical analysis, that the mode of current has a significant effect (*p* < 0.001) on the energy consumptions (EC) during EDBM while the Reynolds number and the coupled effect Reynolds number/mode of current had no effect (*p* > 0.05). The curves calculated for the EC as a function of Reynolds number (Figure 8) showed quite similar values according to standard deviations, but different for the mode of current considered (CC vs PEF). Hence, whatever the Reynolds number, in the case of CC, the value of EC, all Reynolds numbers conditions averaged, was equal to 0.091 ± 0.001 Wh, while it is equal to 0.087 ± 0.001 Wh in the case of PEF. This difference corresponds to a 5% less energy consumption under PEF, which is related to the almost complete absence of protein fouling for the PEF conditions. Slight decrease of EC was also observed by Ruiz et al. [11] during ED of casein solution between conventional ED and PEF with 10–40 s pulses (2.770 Wh and 2.750 Wh respectively under 30 mA/cm^2^) due to the elimination of protein fouling on AEM.

## 4. Conclusions

In this work the influence of solution flow rate (and consequently Reynolds number) and PEF on the protein fouling formation at the cationic interface of BPM (where H^+^ are electrogenerated) and on membrane properties during EDBM of skim milk were demonstrated. It was observed that the major impact on the presence or not of a protein fouling at the BPM cationic interface is the mode of current used while flow rate was less impacting. It can be concluded from obtained results that the application of a PEF mode of current prevented the fouling formation almost completely during EDBM, regardless of the flow rate due to the decrease of CP and less intense H^+^ generation. Flow rate did not have a major impact during application of PEF with very low amount or complete absence of protein fouling while during CC, the weight of fouling was different according to the flow rate applied. The fouling formation under CC was due to the local pH changes at the BPM interface. When a CC mode was applied, a Reynolds number of 323 and over would be the optimal regime in these experimental conditions to minimize the protein fouling of the BPM during EDBM allowing to wash off part of the protein sediment from the membrane surface. CEM showed a decrease in conductivity after EDBM treatment due to the replacement of mobile single charged ions by double charged ions from milk. BPM which was in contact with milk solution also showed a decrease in conductivity due to the protein fouling formation, while conductivity of BPM2 did not change after all treatments. It was observed that EDBM process did not influence the membrane thickness. Using PEF leads to a 5% less EC in comparison with CC regime regardless of Reynolds number considered which is connected with an absence of protein fouling on the BPM membrane surface. 

The next steps, currently underway, are to optimize PEF by testing different pulse–pause duration to find the optimal condition of this current regime and to model the EDBM process for a better understanding of protein fouling kinetics.

## Figures and Tables

**Table 1:** Average composition of milk.

Fat PLS ^1^, % *w*/*w*	Protein, % *w*/*w*	Lactose, % *w*/*w*	Solids, % *w*/*w*	SNF ^2^, % *w*/*w*	Casein, g/L	NPN/CU ^3^, mg/100g
0.13 ± 0.01	3.37 ± 0.05	4.73 ± 0.05	9.14 ± 0.09	8.34 ± 0.09	27.07 ± 0.36	15.60 ± 1.72

^1^ Phospholipids. ^2^ Solids not fat (SNF=Total solids – Fat). ^3^ Non Protein Nitrogen and Calculated Urea.

**Table 2:** Membrane properties (thickness and conductivity) before and after EDBM carried out in different conditions of flow rate (corresponding to indicated Reynolds numbers) and current mode. Reynolds numbers of 162, 242, 323, 404 and 485 correspond to flow rates of 400, 600, 800, 1000 and 1200 mL/min respectively. CC—constant current, PEF—pulsed electric field, CEM—cation exchange membrane and BPM—bipolar membrane.

Current Mode			CC					PEF				
Reynolds Number			162	242	323	404	485	162	242	323	404	485
Conductivity (mS/cm)	CEM	Before	8.35 ± 0.20 a*	8.68 ± 0.30 a	8.49 ± 0.37 a	8.33 ± 0.41 a	8.53 ± 0.22 a	8.33 ± 0.33 a	8.35 ± 0.67 a	8.34 ± 0.69 a	8.02 ± 0.45 a	8.54 ± 0.58 a
		After	7.01 ± 0.17 b	7.41 ± 0.13 b	7.14 ± 0.27 b	7.16 ± 0.30 b	7.30 ± 0.51 b	7.25 ± 0.34 b	6.99 ± 0.45 b	7.04 ± 0.69 b	7.33 ± 0.20 b	7.30 ± 0.23 b
	BPM1	Before	7.59 ± 0.36 a	7.35 ± 0.40 a	7.44 ± 0.46 a	7.40 ± 0.18 a	6.84 ± 1.09 a	7.36 ± 0.21 a	7.14 ± 0.38 a	7.32 ± 0.14 a	7.82 ± 0.15 a	7.53 ± 0.17 a
		After	6.67 ± 0.46 b	6.55 ± 0.34 b	6.74 ± 0.57 b	6.70 ± 0.14 b	6.53 ± 0.30 b	6.58 ± 0.15 b	6.05 ± 0.65 b	6.35 ± 0.41 b	6.75 ± 0.22 b	6.87 ± 0.44 b
	BPM2	Before	7.39 ± 0.31 a	7.35 ± 0.23 a	7.57 ± 0.64 a	7.66 ± 0.46 a	7.54 ± 0.34 a	7.28 ± 0.18 a	7.39 ± 0.33 a	7.17 ± 0.08 a	7.48 ± 0.32 a	7.69 ± 0.07 a
		After	7.11 ± 0.46 bA	7.04 ± 0.39 bA	7.65 ± 0.48 bA	7.51 ± 0.46 bA	7.18 ± 0.09 bA	6.90 ± 0.06 bB	6.79 ± 0.27 bB	6.99 ± 0.34 bB	7.29 ± 0.45 bB	7.07 ± 0.47 bB
Thickness (mm)	CEM	Before	0.151 ± 0.005 a	0.149 ± 0.004 a	0.151 ± 0.006 a	0.144 ± 0.005 a	0.153 ± 0.004 a	0.151 ± 0.005 a	0.151 ± 0.004 a	0.148 ± 0.005 a	0.152 ± 0.006 a	0.143 ± 0.005 a
		After	0.150 ± 0.004 a	0.150 ± 0.004 a	0.148 ± 0.005 a	0.146 ± 0.010 a	0.149 ± 0.003 a	0.149 ± 0.002 a	0.148 ± 0.006 a	0.144 ± 0.004 a	0.155 ± 0.004 a	0.145 ± 0.005 a
	BPM1	Before	0.244 ± 0.004 a	0.244 ± 0.011 a	0.246 ± 0.002 a	0.247 ± 0.003 a	0.246 ± 0.003 a	0.247 ± 0.002 a	0.242 ± 0.006 a	0.246 ± 0.005 a	0.251 ± 0.001 a	0.249 ± 0.005 a
		After	0.240 ± 0.003 b	0.243 ± 0.004 b	0.244 ± 0.003 b	0.238 ± 0.004 b	0.240 ± 0.003 b	0.242 ± 0.003 b	0.243 ± 0.001 b	0.242 ± 0.002 b	0.246 ± 0.003 b	0.245 ± 0.001 b
	BPM2	Before	0.248 ± 0.004 a	0.245 ± 0.005 a	0.247 ± 0.007 a	0.247 ± 0.007 a	0.247 ± 0.005 a	0.246 ± 0.002 a	0.244 ± 0.004 a	0.241 ± 0.002 a	0.243 ± 0.004 a	0.247 ± 0.004 a
		After	0.241 ± 0.004 b	0.241 ± 0.003 b	0.245 ± 0.006 b	0.238 ± 0.004 b	0.245 ± 0.003 b	0.241 ± 0.002 b	0.238 ± 0.010 b	0.241 ± 0.001 b	0.243 ± 0.003 b	0.242 ± 0.002 b

* Data with different letters (a, b or A, B) are significantly different; lowercase letters indicate differences between conductivities and thicknesses before and after EDBM treatment for the same membrane; uppercase letters indicate differences between modes of current for the same membranes.

## References

[1] Dominguez-Salas P., Galie A., Omore A., Omosa E., Ouma E., Ferranti P., Berry E.M., Anderson J.R. (2019). Contributions of Milk Production to Food and Nutrition Security. Encyclopedia of Food Security and Sustainability.

[2] Gorbatova K.K., Gunkova P.I. (2012). Chemistry and Physics of Milk and Dairy Products.

[3] Mier M.P., Ibanez R., Ortiz I. (2008). Influence of process variables on the production of bovine milk casein by electrodialysis with bipolar membranes. Biochem. Eng. J..

[4] Audic J.-L., Chaufer B., Daufin G. (2003). Non-food applications of milk components and dairy co-products: A review. Le Lait..

[5] Van der Horst H.C., Timmer J.M.K., Robbertsen T., Leenders J. (1995). Use of nanofiltration for concentration and demineralization in the dairy industry: Model for mass transport. J. Membr. Sci..

[6] Houldsworth D.W. (1980). Demineralization of whey by means of ion-exchange and electrodialysis. J. Soc. Dairy Technol..

[7] Bazinet L., Lamarche F., Ippersiel D., Amiot J. (1999). Bipolar membrane electroacidification to produce bovine milk casein isolate. J. Agric. Food Chem..

[8] Thompson D.W., Tremblay A.Y. (1983). Fouling in steady and unsteady state electrodialysis. Desalination.

[9] Bleha M., Tishchenko G., Šumberová V., Kůdela V. (1992). Characteristic of the critical state of membranes in ED-desalination of milk whey. Desalination.

[10] Mikhaylin S., Bazinet L. (2016). Fouling on ion-exchange membranes: Classification, characterization and strategies of prevention and control. Adv. Colloid Interface Sci..

[11] Ruiz B., Sistat P., Huguet P., Pourcelly G., Araya-Farias M., Bazinet L. (2007). Application of relaxation periods during electrodialysis of a casein solution: Impact on anion-exchange membrane fouling. J. Membr. Sci..

[12] Dufton G., Mikhaylin S., Gaaloul S., Bazinet L. (2019). Positive Impact of Pulsed Electric Field on Lactic Acid Removal, Demineralization and Membrane Scaling during Acid Whey Electrodialysis. Int. J. Mol. Sci..

[13] Mikhaylin S., Nikonenko V., Pismenskaya N., Pourcelly G., Choi S., Kwon H.J., Han J., Bazinet L. (2016). How physico-chemical and surface properties of cation-exchange membrane affect membrane scaling and electroconvective vortices: Influence on performance of electrodialysis with pulsed electric field. Desalination.

[14] Nikonenko V.V., Pismenskaya N.D., Belova E.I., Sistat P., Huguet P., Pourcelly G., Larchet C. (2010). Intensive current transfer in membrane systems: Modelling, mechanisms and application in electrodialysis. Adv. Colloid Interface Sci..

[15] Sistat P., Huguet P., Ruiz B., Pourcelly G., Mareev S.A., Nikonenko V.V. (2015). Effect of pulsed electric field on electrodialysis of a NaCl solution in sub-limiting current regime. Electrochim. Acta.

[16] Mikhaylin S., Sion A.-V. (2016). Improvement of a sustainable hybrid technology for caseins isoelectric precipitation (electrodialysis with bipolar membrane/ultrafiltration) by mitigation of scaling on cation-exchange membrane. Innov. Food Sci. Emerg. Technol..

[17] Lee H.-J., Sarfert F., Strathmann H., Moon S.-H. (2002). Designing of an electrodialysis desalination plant. Desalination.

[18] Muehlhoff E., Bennett A., McMahon D. (2013). Milk and Dairy Products in Human Nutrition.

[19] Cifuentes-Araya N., Pourcelly G., Bazinet L. (2011). Impact of pulsed electric field on electrodialysis process performance and membrane fouling during consecutive demineralization of a model salt solution containing a high magnesium/calcium ratio. J. Colloid Interface Sci..

[20] Langevin M.-E., Bazinet L. (2011). Ion-exchange membrane fouling by peptides: A phenomenon governed by electrostatic interactions. J. Membr. Sci..

[21] Tanaka Y. (2007). Ion Exchange Membranes: Fundamentals and Applications.

[22] Buchanan J.H., Peterson E.E. (1927). Buffers of milk and buffer value. J. Dairy Sci..

[23] Lucey J.A., Horne D.S. (2009). Milk Salts: Technological Significance. Advanced Dairy Chemistry.

[24] Le Graet Y., Brulé G. (1993). Les équilibres minéraux du lait: Influence du pH et de la force ionique. Lait.

[25] Law A.J.R., Leaver J. (1998). Effects of acidification and storage of milk on dissociation of bovine casein micelles. J. Agric. Food Chem..

[26] Attia H., Kherouatou N., Ayadi J. (2000). Acidification chimique directe du lait: Correlation entre la mobilité du matériel micellaire et les micro et macrostructures des laits acidifiés. Sci. Aliment..

[27] Van Hooydonk A.C.M., Hagerdoorn H.G., Boerrigter I.J. (1986). pH-induced physicochemical changes of casein micelles in milk and their effect on renneting. I. Effect of acidification on physichochemical properties. Neth. Milk Dairy J..

[28] Bazinet L., Lamarche F., Ippersiel D., Gendron C., Mahdavi B., Amiot J. (2000). Comparison of electrochemical and chemical acidification of skim milk. J. Food Sci..

[29] Masson F.-A., Mikhaylin S., Bazinet L. (2018). Production of calcium- and magnesium-enriched caseins and caseinates by an ecofriendly technology. J. Dairy Sci..

[30] Bazinet L., Montpetit D., Ippersiel D., Amiot J., Lamarche F. (2001). Identification of Skim Milk Electroacidification Fouling: A Microscopic Approach. J. Colloid Interface Sci..

[31] Lemay N., Mikhaylin S., Bazinet L. (2019). Voltage spike and electroconvective vortices generation during electrodialysis under pulsed electric field: Impact on demineralization process efficiency and energy consumption. Innov. Food Sci. Emerg..

[32] Salaun M.S., Mietton B., Gaucheron F. (2005). Buffering capacity of dairy products. Int. Dairy J..

[33] Bazinet L., Pouliot Y., Castaigne F. (2010). Relative contributions of charged species to conductivity changes in skim milk during electrochemical acidification. J. Memb. Sci..

[34] Bazinet L., Ippersiel D., Gendron C., Beaudry J., Mahdavi B., Amiot J., Lamarche F. (2000). Cationic balance in skim milk during bipolar membrane electroacidification. J. Memb. Sci..

[35] Lin Teng Shee F., Arul J., Brunet S., Bazinet L. (2008). Performing a three-step process for conversion of chitosan to its oligomers using a unique bipolar membrane electrodialysis system. J. Agric. Food Chem..

[36] Kravtsov V., Kulikova I., Mikhaylin S., Bazinet L. (2020). Alkalinization of acid whey by means of electrodialysis with bipolar membranes and analysis of induced membrane fouling. J. Food Eng..

[37] Bazinet L., Montpetit D., Ippersiel D., Mahdavi B., Amiot J., Lamarche F. (2003). Neutralization of hydroxide generated during skim milk electroacidification and its effect on bipolar and cationic membrane integrity. J. Memb. Sci..

[38] Campione A., Gurreri L., Ciofalo M., Micale G., Tamburini A., Cipollina A. (2018). Electrodialysis for water desalination: A critical assessment of recent developments on process fundamentals, models and applications. Desalination.

